# Metagenomic Analysis of Antibiotic-Induced Changes in Gut Microbiota in a Pregnant Rat Model

**DOI:** 10.3389/fphar.2016.00104

**Published:** 2016-04-28

**Authors:** Imran Khan, Esam I. Azhar, Aymn T. Abbas, Taha Kumosani, Elie K. Barbour, Didier Raoult, Muhammad Yasir

**Affiliations:** ^1^Biochemistry Department, Faculty of Science, King Abdulaziz UniversityJeddah, Saudi Arabia; ^2^Special Infectious Agents Unit, King Fahd Medical Research Center, King Abdulaziz UniversityJeddah, Saudi Arabia; ^3^Medical Laboratory Technology Department, Faculty of Applied Medical Sciences, King Abdulaziz UniversityJeddah, Saudi Arabia; ^4^Biotechnology Research Laboratories, Gastroenterology Surgery Center, Mansoura UniversityMansoura, Egypt; ^5^Biochemistry Department, Faculty of Science – Production of Bioproducts for Industrial Applications Research Group – Experimental Biochemistry Unit, King Fahd Medical Research Center King Abdulaziz UniversityJeddah, Saudi Arabia; ^6^Faculty of Agricultural and Food Sciences, American University of BeirutBeirut, Lebanon; ^7^Adjunct to Biochemistry Department, Faculty of Science – Production of Bioproducts for Industrial Applications Research Group, King Abdulaziz UniversityJeddah, Saudi Arabia; ^8^URMITE CNRS-IRD 198 UMR 6236, Faculté de Médecine et de Pharmacie, Université de la MéditerranéeMarseille, France

**Keywords:** gut microbiota, azithromycin, amoxicillin, cefaclor, pregnancy, rats, 16S rRNA gene

## Abstract

Food and Drug Administration (FDA, USA)-approved category B antibiotics are commonly prescribed to treat infections during pregnancy. The aim of this study was to investigate antibiotic-induced changes in gut microbiota (GM) that occur during pregnancy. The 16S rRNA amplicon deep-sequencing method was used to analyze the effect of category B antibiotics (azithromycin, amoxicillin and cefaclor) on GM during pregnancy using a rat model. The GM composition was substantially modulated by pregnancy and antibiotics administration. Firmicutes, Bacteroidetes, Proteobacteria, Chlamydiae, Actinobacteria, and Cyanobacteria were the dominant phyla. Antibiotic treatment during pregnancy increased the relative abundance of Proteobacteria and reduced Firmicutes. The genera *Shigella*, *Streptococcus*, *Candidatus Arthromitus,* and *Helicobacter* were significantly (*p* < 0.05) more abundant during pregnancy. Antibiotics significantly (*p* < 0.05) reduced the relative abundance of *Lactobacillus* but increased that of *Enterobacter*. There was a significant (*p* < 0.05) decrease in *Lactobacillu*s sp., *Lactobacillus gallinarum* and *Lactobacillus crispatus* during pregnancy. Antibiotic treatment reduced bacterial diversity; the lowest number of operational taxonomic units (OTUs) were detected in the cefaclor-treated groups. Antibiotics significantly (*p* < 0.05) promoted weight gain during pregnancy, and increased relative abundance of *Shigella sonnei*, *Enterococcus hormaechei,* and *Acinetobacter* sp. GM perturbations were accompanied by increases in Proteobacteria abundance and weight gain in pregnancy following antibiotic treatment.

## Introduction

The gut microbiota (GM) is comprised of mutualistic microorganisms that reside in the host gut (Dethlefsen and Relman, 2011). The majority of members of the human GM are bacteria representing six dominant phyla: Firmicutes, Bacteroidetes, Proteobacteria, Verrucomicrobia, Actinobacteria, and Fusobacteria (Ubeda and Pamer, 2012; Khan et al., 2014). These microorganisms play a crucial role in human digestion, metabolism and immunity (Dethlefsen and Relman, 2011; Ferreira et al., 2011). Several studies have associated alterations in GM composition with serious health problems such as diabetes, obesity, allergy, infection and inflammation (Bulloch and Carroll, 2012; Ubeda and Pamer, 2012; Khan et al., 2014). GM composition is plastic in nature and changes greatly with age, diet, medication and physiological status (Dethlefsen and Relman, 2011; Koren et al., 2012). Pregnant women were recently reported to have an abnormal GM composition that changes with trimester (Koren et al., 2012). Overall, GM diversity and richness decrease with pregnancy (Koren et al., 2012). Pregnancy-induced GM changes have been found to correlate with metabolic disorder-like symptoms as demonstrated in a study in which the intestines of germ-free mice were colonized with the GM of pregnant women (Koren et al., 2012). Increases in the densities of Proteobacteria and Actinobacteria during the third trimester of pregnancy were found to be correlated with energy loss in the stool (Koren et al., 2012; Hu et al., 2013). Moreover, the density of beneficial bacteria, such as *Faecalibacterium*, which has anti-inflammatory properties, is significantly reduced during pregnancy (Koren et al., 2012). GM perturbations during pregnancy increase the risk of infection, which can lead to serious illnesses such as digestive problems and miscarriages (Amann et al., 2006). These recent findings have motivated researchers to investigate the associations between GM and human health.

In past decades, it was assumed that the gastrointestinal problems experienced by pregnant women, such as diarrhea, hemorrhoids, stomach ache, acidity, headache, and vomiting, were typical normal features of pregnancy. However, recent studies have associated these symptoms with dysbiotic GM and increasing risk of infection (Poveda et al., 2014; Shaban et al., 2014). Poveda et al. (2014) observed that hyperemesis, dyspepsia and gastric discomfort during pregnancy were associated with *Helicobacter pylori* infection. It has been observed that many pregnant women suffering from inflammatory bowel disease (IBD) have lower bacterial diversity and a decreased density of gut Enterobacteriaceae relative to pregnant women without IBD (Poveda et al., 2014). A recent survey reported that 800 women die every day due to pregnancy complications, including gastrointestinal infections (Say et al., 2014). Being immune-compromised, pregnant women are more susceptible to opportunistic pathogens (Petersen et al., 2010; Poveda et al., 2014). The most common bacterial pathogens that induce acute diarrhea in pregnant women are *Shigella* sp., *Campylobacter* sp., *Yersinia* sp., *Escherichia coli,* and *Salmonella* sp. (Wald, 2003). A study using a non-human animal model found that infections during pregnancy induced neuropathologies and altered behavior (Amann et al., 2006; Hu et al., 2013). In addition, the rapid increase in antibiotic resistance is reducing the physician’s range of antibiotics that are considered safe during pregnancy (Petersen et al., 2010; Hu et al., 2013). Currently, physicians are mainly prescribing B-category antibiotics that are recommended by the FDA, which are considered to be non-teratogenic (Petersen et al., 2010). In particular, increases in the use of beta-lactam antibiotics, e.g., penicillin and cephalosporin, during pregnancy have been reported (Amann et al., 2006; Petersen et al., 2010). Amoxicillin is the most commonly used antibiotic during pregnancy (Amann et al., 2006; Petersen et al., 2010). Several studies have reported modulation in GM-associated phyla (Actinobacteria, Firmicutes, Bacteroidetes, and Proteobacteria) with broad-spectrum antibiotic treatment (Ubeda et al., 2010; Claesson et al., 2011; Murphy et al., 2013). Alterations of GM during pregnancy could lead to pregnancy complications. The aim of this study was to determine the impacts of pregnancy and the antibiotics amoxicillin, cefaclor and azithromycin, which are widely used during pregnancy, on GM in a rat model. Total metagenomic DNA was extracted from rat intestines and then subjected to 16S amplicon deep sequencing to characterize the changes in GM diversity and richness.

## Methodology

### Rat Model and Sample Collection

The research ethics committee of the Faculty of Medicine at King Abdulaziz University approved the study protocol under agreement number (HA-02-J-008). The experiment was carried out in accordance with approved guidelines. Forty-eight sexually mature, female Sprague–Dawley rats that were 8 weeks old and weighed approximately 200–250 g were obtained from the animal breeding unit of the King Fahd Medical Research Center at King Abdulaziz University Jeddah, Saudi Arabia. The rats were acclimatized to standardized laboratory conditions at a temperature of 21 ± 1°C, a relative humidity of 50 ± 10% and a 12-h light–dark cycle. These environmental parameters were maintained throughout the study. One week before conception, the rats were housed in polycarbonate cages with hardwood chip bedding. Conception for pregnant rats was confirmed through detection of a vaginal plug. The first detection of sperm on the plugs marked day D-0 of gestation. Rats have an average gestational period of 21 days (Awodele et al., 2012). Antibiotics suspensions were orally administered to rats on the 13th day of gestation because the first 12 days of rat pregnancy are critical for embryogenesis (Zalgevičienė, 2010; Er, 2013). The antibiotics doses were selected based on human adult dosage, and converted to rat dosage using body weight Ghosh’s formula. The following dose of amoxicillin (50 mg/kg/day), azithromycin (45 mg/kg/day) or cefaclor (67.5 mg/kg/day) was administered by oral gavage in water suspension for 7 days (Ghosh, 2007; Gottberg et al., 2014). Rats were divided into eight groups based on antibiotic treatment and pregnancy: non-pregnant control (NPC) rats, pregnant control rats (PC), non-pregnant rats treated with azithromycin (NPAzi), pregnant rats treated with azithromycin (PAzi), non-pregnant rats treated with amoxicillin (NPAmx), pregnant rats treated with amoxicillin (PAmx), non-pregnant rats treated with cefaclor (NPCef) and pregnant rats treated with cefaclor (PCef, Supplementary Figure S1). Each group comprised six rats. All rats were sacrificed on the 20th day of gestation, before delivery. The intestinal sections were collected in sterile tubes. The samples were stored at -80°C for DNA extraction.

### 16S rRNA Gene Sequencing and Data Processing

Total metagenomic DNA was extracted from the intestinal section using QIAamp Fast DNA Stool Mini Kit (Qiagen, USA). The samples were sequenced for 16S rRNA genes targeting the V4 region with barcoded 515F GTGCCAGCMGCCGCGGTAA and 806R GGACTACVSGGGTATCTAAT universal primers following the procedure of Dowd et al. (2008). DNA concentrations were determined using the Qubit system (Invitrogen, USA). Subsequently, dual-index barcodes and Illumina sequencing adapters were joined to the reads using a limited PCR cycle. After purification with Agencourt AMPure beads (Agencourt, USA), libraries were normalized using the Nextera XT protocol. Samples were pooled into a single flow cell for sequencing on the MiSeq sequencing platform (Illumina, USA) following the manufacturer’s protocol. Automated cluster generation and paired-end sequencing with dual index reads were performed in a single run with a read length of 2 × 300 bp.

Raw FASTQ files were obtained from the Illumina MiSeq, and paired end reads were collected using PANDAseq (Masella et al., 2012). Sequences were cleaned of primers and barcodes. Additionally, all reads with ‘N’ and those with sequences <200 bp were deleted, and high-quality sequences were dereplicated (Boissière et al., 2012). The cleaned sequences were then clustered at *k* = 10 (97% similarity) followed by deletion of chimeras and singleton reads (Eren et al., 2011; Swanson et al., 2011). Finally, operational taxonomic units (OTUs) were classified using BLASTn against a curated database derived from GreenGenes, RDPII and NCBI. Sequence data of this study is submitted in the NCBI under project no. PRJNA300497.

### Statistical Analysis

The OTU biodiversity and richness were calculated using R package phyloseq (1.7.24) implemented with non-parametric Chao1 and rarefaction analysis that showed the uniformity and distribution of OTUs in different groups (McMurdie and Holmes, 2012). R package “rich” (0.3) was employed for statistical modulations in specie richness and abundance. The Kolmogorov–Smirnov *D* test was used to ascertain the normality of the data. One-way ANOVA (for parametric data) and non-parametric Kruskal–Wallis and Mann–Whitney tests (for non-normal data) were performed to identify significantly different bacterial taxa among different groups (Supplementary Figure S1). SPSS version 22 was used for statistical analysis. The VassarStats website^1^ was used to conduct pair-wise comparisons of rat weight via the Tukey’s HSD test. The Shannon index was calculated using the R statistical framework version 3.1.2 with VEGAN package version 2.2-1.

## Results

### Common Core Bacteria

**FIGURE 1:**
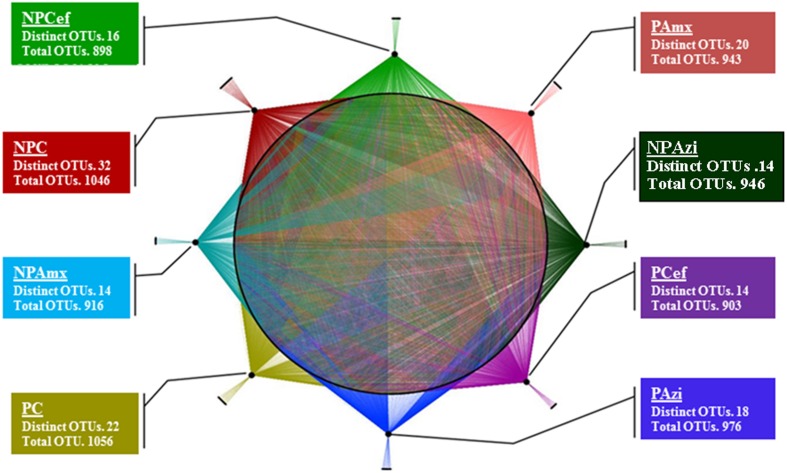
**Operational taxonomic units (OTUs) presentation and analysis at specie level.** Network presentation of OTUs at the species level among the groups. Species shared among different groups are connected by multiple threads, whereas unique bacterial OTUs are connected to their respective groups by a single line. NPC, non-pregnant control; PC, pregnant control; NPAzi, non-pregnant rats exposed to azithromycin; PAzi, pregnant rats exposed to azithromycin; NPAmx, non-pregnant rats exposed to amoxicillin; PAmx, pregnant rats exposed to amoxicillin; NPCef, non-pregnant rats exposed to cefaclor; PCef, pregnant rats exposed to cefaclor.

In total, 5.7 million raw sequence reads were obtained using the MiSeq system (Illumina, San Diego, CA, USA). After filtration, approximately 5.6 million high quality (>250 bp) sequence reads were obtained and assigned to bacterial domain. Overall, 44 phyla were identified in the entire model system. Thirty-three phyla were commonly found in each group, and 10 phyla were dominant at ≥1% concentration but varied in relative abundance with pregnancy and antibiotic treatment. The majority of sequencing reads were representative of the phyla Firmicutes, Proteobacteria and Bacteroidetes, which dominated the bacterial community in each of the studied groups. A total of 292 families were detected. Among them, 120 families were commonly present, and Clostridiaceae, Lactobacillaceae, Helicobacteraceae, Streptococcaceae, and Bacillaceae were the dominant families detected. A total 788 OTUs were identified at the genus level. The highest number of genera (591) was detected in pregnant control (PC), whereas the lowest (505) was found in non-pregnant rats treated with cefaclor (NPCef). Antibiotics significantly decreased the number of genera in non-pregnant rats treated with azithromycin (NPAzi, *p* = 0.008, 529), non-pregnant rats treated with amoxicillin (NPAmx, *p* = 0.012, 525) and NPCef (*p* = 0.002, 506) relative to the number in NPC. The *Clostridium* and *Shigella* were the dominant genera associated with pregnancy. We observed 1466 different species; approximately 1316 species were shared in common among the rat groups, and 150 were species unique to one or more groups. The highest number of species was observed in PC (1056) followed by NPC (1046). The lowest species diversity was observed in pregnant rats treated with cefaclor (PCef, 903) (**Figure 1**). Species diversity substantially decreased with antibiotic treatment in both pregnant and non-pregnant rat groups. In particular, significantly decreased (*p* = 0.05; *p* = 0.018) species diversity was observed among cefaclor treated pregnant and non-pregnant rats (Supplementary Figure S2). The effect of amoxicillin on OTUs richness was also severe, which was more severe (*p* = 0.036) during pregnancy (PAmx). In term of OTUs diversity, azithromycin was least damaging among the tested antibiotics. Among the dominant bacterial species, *Lactococcus lactis*, *Bacillus thermoamylovorans,* and *Clostridium* sp. were commonly abundant in all groups.

### Gut Microbiota Differences between Pregnant and Non-pregnant Control Groups

**FIGURE 2:**
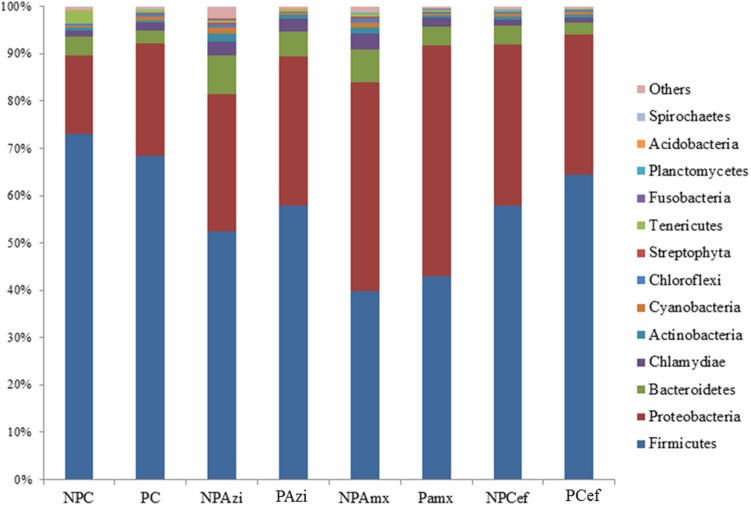
**Average relative density of the dominant bacterial phyla.** The *x*-axis shows the taxon groups, and the *y*-axis displays the average percentages of sequences reads. The cutoff point for selecting the dominant phyla was set to ≥1%. “Others” indicates minor phyla. Abbreviations are as described for **Figure 1**.

**FIGURE 3:**
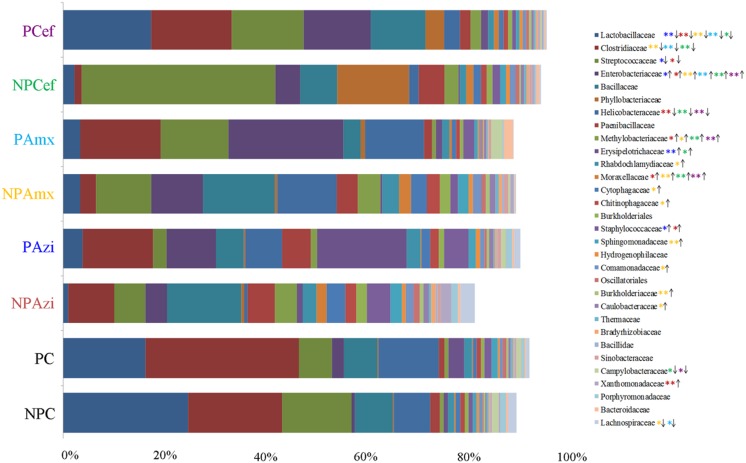
**Comparative analysis of the dominant and significantly different families in the gut microbiota of control versus pregnant and antibiotic-treated rat groups.** The densities are shown in average percentage. The direction of arrow is indicating increase or decrease in the density of each family in compare to respective control. The color of steric represents significant changes in the respective group. ^∗^Indicates significant at *p* < 0.05. ^∗∗^Indicates highly significant at *p* ≤ 0.01. Abbreviations are as described for **Figure 1**.

**FIGURE 4:**
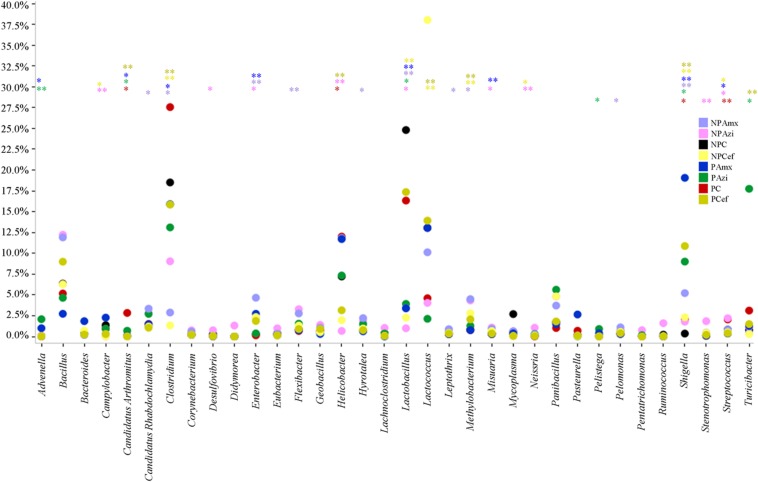
**Comparative analysis of the dominant and significantly different genera in the gut microbiota of control versus pregnant and antibiotic-treated rat groups.** The color of steric (^∗^) represents significant change in the respective groups. ^∗^Indicates significant at *p* < 0.05. ^∗∗^Indicates highly significant at *p* ≤ 0.01. Abbreviations are as described for **Figure 1**.

**FIGURE 5:**
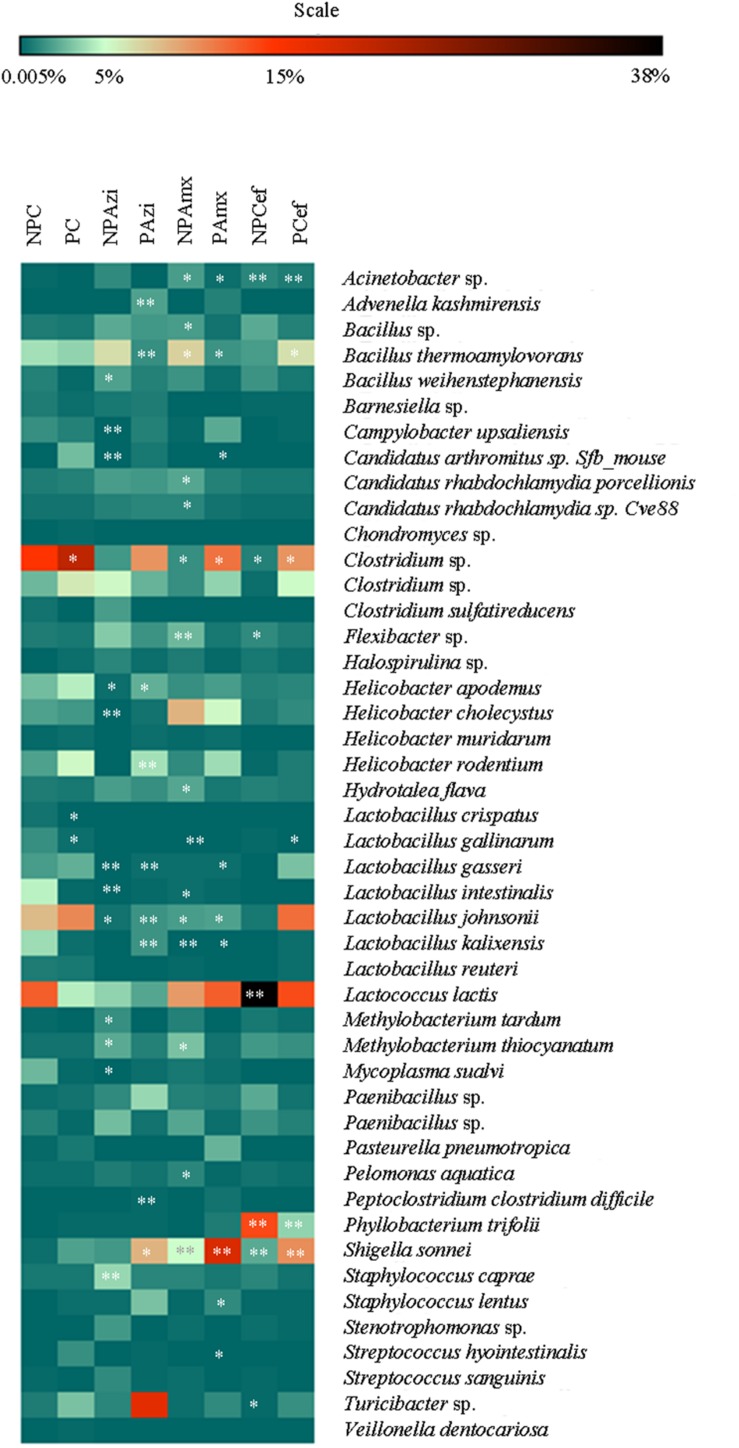
**Heat map of the dominant and significantly different species in the gut microbiota of control versus pregnant and antibiotic-treated rat groups.** The stearic represents significant change in respective group. ^∗^Indicates significant at *p* < 0.05. ^∗∗^Indicates highly significant at *p* ≤ 0.01. Abbreviations are as described for **Figure 1**.

Percentage of the dominant phyla modulated with pregnancy however the change was statistically insignificant. Percentage relative abundance of the phyla Firmicutes, Bacteroidetes, and Tenericutes was decreased in PC compared with NPC. The density of Proteobacteria was increased in pregnant rats relative to non-pregnant rats (**Figure 2**). The GM of the NPC group was predominantly composed of the families Lactobacillaceae (24.9 ± 5.48%), Clostridiaceae (18.6 ± 4.6%), Streptococcaceae (13.9 ± 5.2%), Bacillaceae (7.5 ± 5.4%) and Helicobacteraceae (7.2 ± 2.7%). Comparatively, the density of Clostridiaceae (30.4 ± 8.6%), Helicobacteraceae (12 ± 6.75%), Erysipelotrichaceae (3.1 ± 1.5%) and Enterobacteriaceae (14.3 ± 3.6%) was increased in PC. The concentrations of Lactobacillaceae (16.4 ± 7.5%) and Streptococcaceae (6.64 ± 2.3%) were decreased in PC relative to the corresponding values in NPC (**Figure 3**). *Shigella* (2.1 ± 0.55%), *Streptococcus* (2.06 ± 0.89%), *Candidatus Arthromitus* (0.9 ± 0.06%) and *Helicobacter* (12 ± 3.6%) abundance significantly (*p* < 0.05) increased with pregnancy relative to NPC (**Figure 4**). *Lactobacillus* was the dominant genus (24%) in NPC and comprised 27 species. *Clostridium* sp. (21.09 ± 8.02) significantly (*p* = 0.04) increased in relative abundance with pregnancy, and the concentrations of *Helicobacter rodentium* (5.4 ± 1.13), *Turicibacter* sp. (3.1 ± 1.3), *Helicobacter apodemus* (4.49 ± 1.35) and *Streptococcus hyointestinalis* (1.48 ± 0.71) also increased with pregnancy. We observed a significant decline in *Lactobacillus* sp. (*p* = 0.037, 0.79 ± 0.3) and *Lactobacillus gallinarum* (*p* = 0.022, 0.43 ± 0.06) in PC relative to NPC (**Figure 5**). *Lactococcus lactis*, *Lactobacillus johnsonii*, *Shigella sonnei*, *Clostridium* sp., *Turicibacter* sp. and *Helicobacter apodemus* had higher relative abundances in PC than in NPC (**Figure 5**).

### Gut Microbiota Alteration in Response to Azithromycin

In the NPAzi and PAzi groups, azithromycin treatment reduced the density of Firmicutes but increased that of Proteobacteria (**Figure 2**). Azithromycin significantly enriched the families Enterobacteriaceae (*p* = 0.023) and Staphylococcaceae (*p* = 0.031) and decreased that of Streptococcaceae in the NPAzi and PAzi groups. The family Erysipelotrichaceae was significantly (*p* = 0.004) enriched in the PAzi group, and Helicobacteraceae (*p* = 0.001) concentration decreased in NPAzi. Azithromycin significantly decreased the relative abundance of Lactobacillaceae (*p* = 0.0016) in the NPAzi and PAzi groups (**Figure 3**). There were significant increase in abundance of the genera *Turicibacter* (*p* = 0.003) and decrease of *Candidatus Arthromitus* (*p* = 0.002) in PAzi relative to PC, whereas *Lactobacillus* (*p* = 0.002) decreased in both NPAzi and PAzi relative to PC and NPC. Additionally, the genera *Shigella* (*p* = 0.037), *Advenella* (*p* = 0.006) and *Stenotrophomonas* (*p* = 0.009) were significantly enriched in PAzi. Azithromycin significantly decreased *Campylobacter* (*p* = 0.007) and enriched *Mitsuaria* (*p* = 0.035) and *Enterobacter* (0.026) in NPAzi compared with NPC (**Figure 4**).

Azithromycin reduced bacterial species diversity, and the effect was more marked in NPAzi (928), with an 11.2% decrease in OTUs relative to NPC (1046). The total OTU count was 976 in PAzi (**Figure 1**). Moreover, azithromycin caused an increase in the number of dominant species (≥1%) to 19 in both the NPAzi and PAzi groups relative to the NPC and PC groups, which had 15 and 13 dominant species, respectively. Among the dominant species, only *Clostridium* sp., *L. lactis* and *B. thermoamylovorans* were common to both in NPAzi and NPC. *Sphingomonas* sp., *H. cholecystus* and *Streptococcus hyointestinalis* were dominant in the PC group and decreased in PAzi. In addition, NPAzi and PAzi were colonized by 14 and 18 unique bacteria, respectively. We observed significant increases in *Shigella sonnei* (*p* = 0.037), *Advenella kashmirensis* (*p* = 0.006), *Lactobacillus kalixensis* (*p* = 0.007) and *Peptoclostridium clostridium* *difficile* (*p* = 0.001) relative to PC. *Helicobacter apodemus* (NPAzi), *Lactobacillus johnsonii* (NPAzi and PAzi), *Bacillus thermoamylovorans* (PAzi), and *Lactobacillus gasseri* (NPAzi and PAzi) decreased significantly following azithromycin treatment (**Figure 5**). Azithromycin increased *Turicibacter* sp. (17.7 ± 7.5%) in pregnant rats; this taxon had relative abundances of 3.11 ± 1.09% and 2.07 ± 1.02% in NPC and PC, respectively.

### Gut Microbiota Alteration in Response to Amoxicillin

Amoxicillin increased Proteobacteria density and reduced that of Firmicutes in both NPAmx and PAmx (**Figure 2**). The families Clostridiaceae (*p* = 0.009) and Lactobacillaceae (*p* = 0.004) signi ficantly decreased whereas Enterobacteriaceae (*p* = 0.001) increased in both NPAmx and PAmx. We also observed a significant increase in Rhabdochlamydiaceae (*p* = 0.042), Chitinophagaceae (*p* = 0.024), Cytophagaceae (*p* = 0.011), Methylobacteriaceae (*p* = 0.013), Burkholderiales (*p* = 0.01), Comamonadaceae (*p* = 0.007), Caulobacteraceae (*p* = 0.012), and Moraxellaceae (*p* = 0.009) in the NPAmx group (**Figure 3**). Similarly, the genera *Enterobacter* (*p* = 0.003) and *Shigella* (*p* = 0.001) significantly increased, and *Lactobacillus* (*p* = 0.004) and *Clostridium* (*p* = 0.019) decreased in both groups (NPAmx and PAmx) (**Figure 4**). As with azithromycin treatment, a profound decrease in bacterial diversity was observed in the amoxicillin-treated groups NPAmx (12.4%) and PAmx (9.8%) compared with NPC. There were 14 distinct species detected in NPAmx and 20 detected in PAmx (**Figure 1**). The diversity of the dominant species (≥1% concentration) was higher in NPAmx (20 species) than in PAmx (12 species); the corresponding values in NPC and PC were 15 and 13, respectively. Amoxicillin significantly (*p* = 0.001) increased *Shigella sonnei*. Moreover, the dominant species in PAmx were *Lactococcus lactis* and *Clostridium* sp. (**Figure 5**).

### Gut Microbiota Alteration in Response to Cefaclor

The smallest changes in Firmicutes and Proteobacteria density with antibiotic treatment were observed in the cefaclor groups (NPCef and PCef) (**Figure 2**). Cefaclor significantly decreased Helicobacteraceae (*p* = 0.01) and increased Methylobacteriaceae (*p* = 0.009), Enterobacteriaceae (*p* = 0.002) and Moraxellaceae (*p* = 0.003) in both NPCef and PCef (**Figure 3**). In addition, Lactobacillaceae (*p* = 0.008) and Mycoplasmataceae (*p* = 0.045) were significantly reduced in NPCef. We observed a significant (*p* ≤ 0.01) increase in the genera *Lactococcus*, *Shigella*, *Enterobacter,* and *Methylobacterium* in the PCef group (**Figure 4**). Collectively, 30 distinct bacteria species were detected in the NPCef and PCef groups (**Figure 1**). The most dominant species was *Lactococcus* *lactis* in NPCef (38 ± 7.2%), but its relative abundance was reduced to 13.9 ± 4.7% in PCef. Furthermore, the predominant species in PCef were *Lactococcus lactis*, *Clostridium* sp. (10.42 ± 4.23%), *Lactobacillus* *johnsonii* (12.35 ± 2.21%) and *Bacillus thermoamylovorans* (6.68 ± 3.23%) (**Figure 5**).

### Biodiversity and Microbial Richness

**FIGURE 6:**
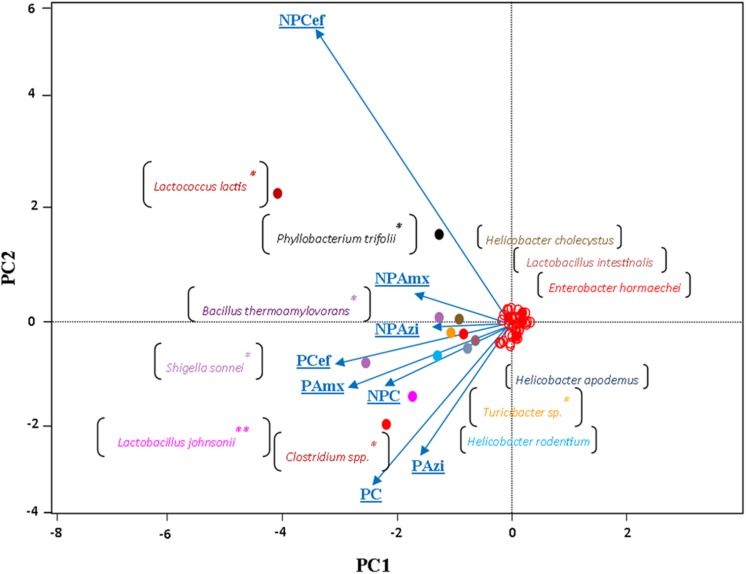
**The multivariate principal coordinate data analysis of the groups using type-1 scaling.** The projecting vectors represent groups and their variability. The colored points labeled with species names show enrichment along respective groups. ^∗^Indicates significant at *p* < 0.05. ^∗∗^Indicates highly significant at *p* ≤ 0.01. Abbreviations are as described for **Figure 1**.

Alpha diversity was estimated through rarefaction analysis, the Shannon index and the Chao1 index. The lowest OTU richness was observed in the cefaclor-treated groups (NPCef and PCef). The highest Chao1 value was observed for PC followed by NPC and PAzi. Rarefaction analysis indicated variation among samples and showed sufficient data coverage for diversity in the samples (Supplementary Figure S3). The Shannon Wiener index, calculated at 3% dissimilarity, showed the lowest value of evenness (2.8) for NPCef. The highest value of evenness was observed for NPAzi (4.4) and NPAmx (4.2). The identified genera from all sequence reads were compared using UPGMA distance matrix clustering analysis. The pregnant groups treated with antibiotics formed a separate cluster from the non-pregnant groups (Supplementary Figure S4). Similarly, the multivariate principal coordinate data analysis separated the pregnant and non-pregnant groups. NPcef showed more divergence than did the other groups (**Figure 6**).

### Antibiotic-Induced Weight Gain and Associated GM Changes

Rats were weighed on D-0 and at the time of sacrifice. The D-0 weight was subtracted from final weight to observe relative weight gain. Among the non-pregnant, antibiotic-treated rat groups, weight gain was significant (*p* = 0.00) between these two time points in the NPCef group (21.4 ± 2.27 g). Among the pregnant groups, significant (*p* < 0.05) weight gain was observed in PAzi (82.3 ± 1.9 g), PAmx (99.3 ± 4.7 g) and PCef (79.1 ± 4.5 g) in comparison with PC (69.5 ± 1.7 g, Supplementary Figure S5). In the pregnant, antibiotic-treated rats, the family Enterobacteriaceae and the species *Shigella sonnei* were the only taxa with significant increases in relative abundance relative to PC. Higher densities of Proteobacteria and Bacteroidetes were detected in pregnant rats treated with antibiotics in comparison to PC (**Figure 2**). Several studies have associated increasing levels of *E. coli* with weight gain (Collado et al., 2008; Chichlowski and Hale, 2009; Evans et al., 2014). We observed that antibiotic treatment promoted the growth of *E. coli* in pregnant rats. Moreover, *Shigella*, which was significantly increased in the antibiotic-treated pregnant rat groups, is very closely related to *Escherichia* and difficult to distinguish from *Escherichia* based on 16S amplicon sequencing.

## Discussion

Pregnancy accompany with GM remodeling that participate in modulating host metabolism and other physiological changes (Koren et al., 2012). These modulations could be supportive in fetal nourishment, however, few in-depth data is published to comprehend GM role in this critical time. In this study, we adopted a 16S rRNA amplicon deep sequencing approach to analyze the antibiotic-induced changes in GM that occur during pregnancy. We observed that majority of dominant taxa were similar with modulated abundances between NPC and PC. The abundance of Firmicutes profoundly impacted during pregnancy that could be related to reduction in the abundance of health promoting families, such as Lactobacillaceae, Streptococcaceae, and Bacillaceae. These taxa have probiotic abilities against various types of diarrhea, functional abdominal pain, ulcerative colitis and digestive problems (Hanning et al., 2010; Aagaard et al., 2012). Dysbiosis in these groups may be one of the reasons behind GIT problems during pregnancy. However, in exception, family Clostridiaceae enriched during pregnancy which is in agreement with Aagaard et al. (2012). Clostridiaceae is butyrate-producing family that improves intestinal lining and help colonocytes in energy extraction. The absence of butyrate can lead to dysfunctional intestinal architecture which could be easily breached by intestinal bacteria and molecules in the bloodstream (Lecomte et al., 2015). Like Koren et al. (2012) we observed substantial reduction in the abundance of Proteobacteria during pregnancy. Proteobacteria enrichment has been associated with increasing inflammation level during pregnancy (Koren et al., 2012). A single member (*E. coli*) of Proteobacteria is sufficient to induce inflammation and disturb glucose and insulin tolerance in germ free mice (Caesar et al., 2012). Several other studies have documented pregnancy-induced changes in GM density and composition, especially the aberrant ratio of Proteobacteria to Firmicutes (De La Cochetiere et al., 2005; Yap et al., 2008; Antonopoulos et al., 2009; Dethlefsen and Relman, 2011). As GM is actively involved in host digestion and metabolism, we suggest in correlating modulated GM composition and richness with metabolomics that could help to understand metabolic complication during pregnancy. Interestingly, based on our literature review using PubMed, this study provides the first detection of the phyla Ignavibacteriae, Thermotogae, Candidatus Saccharibacteria, Dictyoglomi, and Acetothermia in the rat GM. Detection of these phyla could be attributed to the deep 16S amplicon sequencing. Moreover, bacterial diversity enriched during pregnancy in comparison to NPC which could be beneficial in term of GM inheritance to infants; as diversified GM has been associated with lower risks of allergic reactions in infants (Rautava et al., 2012; Zijlmans et al., 2015).

Antibiotics substantially reduced species diversity; as broad-spectrum antibiotics disrupt microbial balance, affecting pathogenic as well as host-associated microbes (Heinsen et al., 2015). The lowest species diversity was observed in the PCef group. Cefaclor is the second-generation cephalosporin with broad spectrum properties (Tomić et al., 2016) against aerobe and anaerbe bacteria (Mangla and Aggarwal, 2012). The wide range of antimicrobial ability of cefaclor may be one of the reasons behind drastic reduction in OTUs diversity in cefaclor treated groups. Amoxicillin also profoundly reduced species diversity, however, the effect was significant during pregnancy. Additionally, the percentage relative abundances of dominant phyla changed following antibiotic treatment compared with their levels in PC and NPC. Moreover, antibiotics effects toward individual species were different. For instance, amoxicillin and azithromycin clearly reduced abundance of *Lactobacillus* *johnsonii.* However, cefaclor enriched *Lactobacillus* *johnsonii* in PCef. *Lactobacillus* *johnsonii* is probiotics bacteria, inhabits the upper part of gastrointestinal tract, actively participates in bile acid hydrolysis and assists the host in combating *Helicobacter* *pylori* infections (Pantoflickova et al., 2003; Aagaard et al., 2012). Antibiotics-induced changes to the composition or metabolism of microbiota may adversely affect pregnancy outcomes and could lead to pregnancy complications (Zhang et al., 2015). Therefore, we suggest screening of other category B antibiotics for GM richness and abundance to ensure minimum damage to beneficial flora.

We noticed that antibiotics treatment enriched the abundance of opportunistic pathogens, especially in genera *Enterobacter*, *Shigella*, *Helicobacter,* and *Clostridium* that could be related to Firmicutes/Proteobacterial aberration. All these modulated taxa are positively correlated with weight gain in pregnant groups. Several others studies have also documented modulated abundances of some of these taxa with weight gain (Cani et al., 2007; Santacruz et al., 2010; Koren et al., 2012). The gram-negative bacteria have been reported to induce weight by elevating endotoxin and inflammatory markers level (Cani et al., 2007; Santacruz et al., 2010). Furthermore, probiotic bacteria, such as *Lactobacillus* species, declined with antibiotics treatment that is inversely correlated with weight gain (Yasir et al., 2015). *L. reuteri* strain ATCC PTA 4659 has the potentials to prevent host from diet-induced obesity (Fak and Backhed, 2012).

In contrast to the findings of Lecomte et al. (2015), we found that the rat gastrointestinal tract was dominated by *Lactobacillus intestinalis*. Similarly, the predominant GM species in the NPC group were *Clostridium* sp. and *Lactococcus* *lactis* followed by *Lactobacillus johnsonii*, *Bacillus* *thermoamylovorans* and *Lactobacillus* *kalixensis*. The differences between the two studies could be due to differences in experimental procedure and sequencing methodology. Since GM composition is plastic in nature and can also fluctuate with geological differences (Quercia et al., 2014). Some of the detected species are water dwelling species, i.e., *Hydrotalea flava* and *Pelomonas aquatica* that enriched with amoxicillin treatment in non-pregnant rats. It is normal to isolate bacteria from unusual habitat, for instance *Pelomonas* has been recently reported in human stomach (Yang et al., 2016).

Overall, antibiotics administration during pregnancy decreased bacterial diversity and promoted weight gain in particular with cefaclor treatment. Relative abundance of beneficial bacteria from Lactobacillus group associated with the host metabolism were decreased, and growth of several opportunistic pathogenic species were increased. Different classes of antibiotics produced varying effects on maternal GM composition during pregnancy and providing potential clues to aid in the clinical selection of antibiotics. In conclusion, antibiotics that are commonly prescribed during pregnancy should be analyzed for microbiome to understand maternal dysbiosis-related body maladaptation and associated pregnancy complications.

## Author Contributions

IK and MY contributed to the experimental work, data analysis and manuscript drafting. EA contributed to the experimental design and manuscript writing. AA performed the animal experiments. TK, DR, and EB critically reviewed the article.

## Conflict of Interest Statement

The authors declare that the research was conducted in the absence of any commercial or financial relationships that could be construed as a potential conflict of interest.

## Footnotes

1 http://vassarstats.net/
