# Supplementary material for: Metagenomic Analysis of Antibiotic-Induced Changes in Gut Microbiota in a Pregnant Rat Model
Source: Front Pharmacol. 2016 Apr 28;7:104. doi: 10.3389/fphar.2016.00104 (PMC4849429; doi:10.3389/fphar.2016.00104)
Supplement: Supplementary file 1 [file Data_Sheet_1.PDF]

Metagenomic analysis of antibiotic-induced changes in gut microbiota in a pregnant rat model

Imran Khan[1,2]†, Esam I. Azhar[2,3], Aymn T. Abbas[2,4], Taha Kumosani[1,5], Elie K. Barbour [6,7], Didier Raoult[2,8] and Muhammad Yasir[2*]†

1 Biochemistry Department, Faculty of Science, King Abdulaziz University, Jeddah, Saudi Arabia,

2 Special Infectious Agents Unit, King Fahd Medical Research Center, King Abdulaziz University, Jeddah, Saudi Arabia,

3 Medical Laboratory Technology Department, Faculty of Applied Medical Sciences, King Abdulaziz University, Jeddah, Saudi Arabia,

4 Biotechnology Research Laboratories, Gastroenterology Surgery Center, Mansoura University, Mansoura, Egypt,

5 Biochemistry Department, Faculty of Science – Production of Bioproducts for Industrial Applications Research Group – Experimental Biochemistry Unit, King Fahd Medical Research Center King Abdulaziz University, Jeddah, Saudi Arabia,

6 Faculty of Agricultural and Food Sciences, American University of Beirut, Beirut, Lebanon,

7 Adjunct to Biochemistry Department, Faculty of Science – Production of Bioproducts for Industrial Applications Research Group, King Abdulaziz University, Jeddah, Saudi Arabia,

8 URMITE CNRS-IRD 198 UMR 6236, Faculté de Médecine et de Pharmacie, Université de la Méditerranée, Marseille, France

*Corresponding Author: Muhammad Yasir**

E-mail:yasirkhattak.mrl@gmail.com

Phone: +966-26401000/25303

Fax: +966-26952076

[+]These authors equally contributed to this work

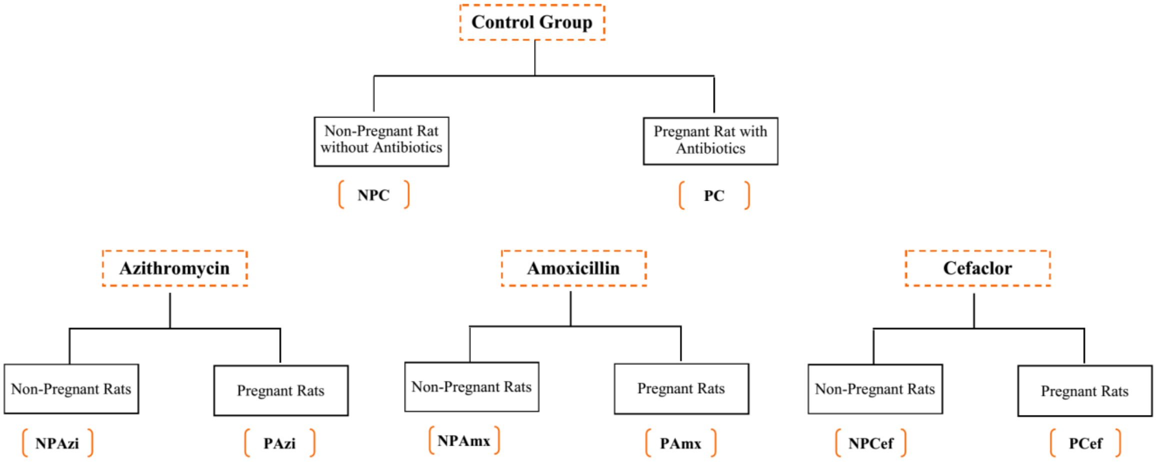

Figure S1: Schematic diagram of experimental model. The alphabets mentioned in brackets represent abbreviations for respective group that used throughout the manuscript. NPC, non-pregnant control; PC, pregnant control; NPAzi, non-pregnant rats exposed to azithromycin; PAzi, pregnant rats exposed to azithromycin; NPAmx, non-pregnant rats exposed to amoxicillin; PAmx, pregnant rats exposed to amoxicillin; NPCef, non-pregnant rats exposed to cefaclor; PCef, pregnant rats exposed to cefaclor

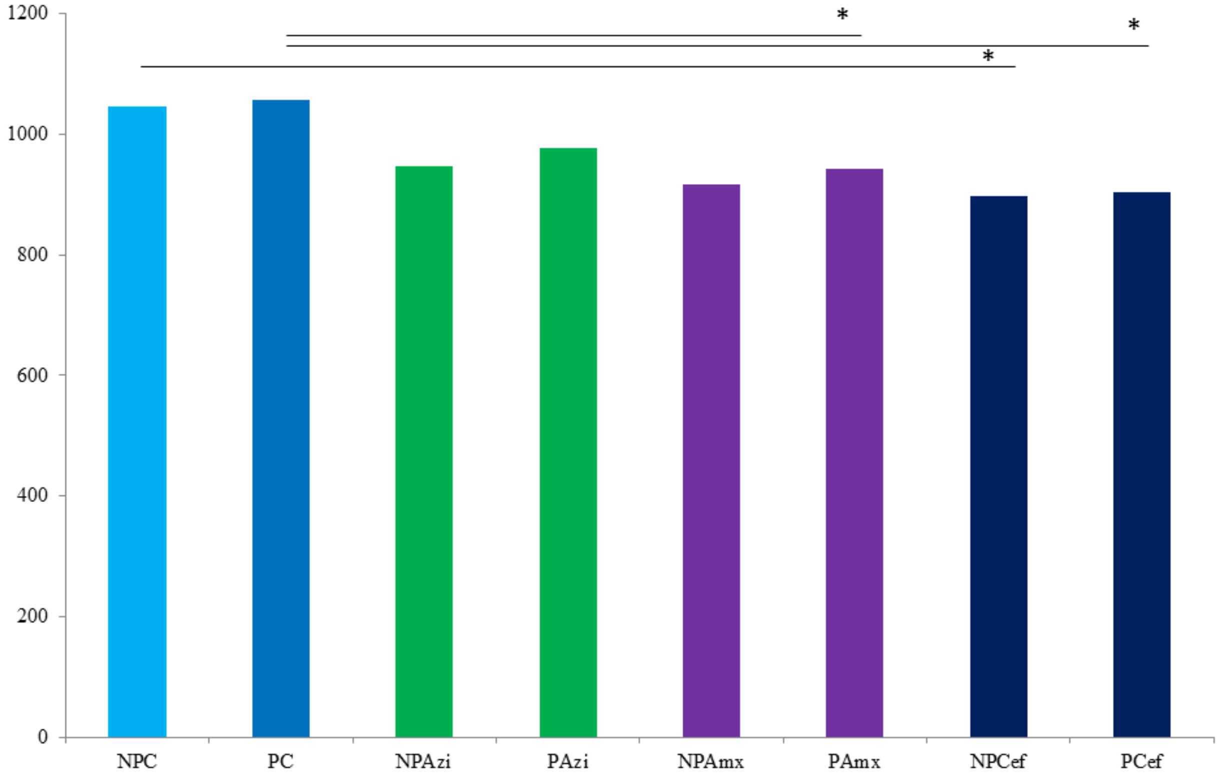

Figure S2: Statistical analysis of OTUs diversity. OTUs count slightly enriched during pregnancy compare to non-pregnant rats. Antibiotics substantially reduced OTUs count. OTUSs diversity significantly decreased in cefaclor treated pregnant (p) and non-pregnant (p) rats in comparison to PC and NPC respectively. The effect of amoxicillin was significant during pregnancy in comparison to PC. The vertical axes is indicating OTUs measure. NPC, non-pregnant control; PC, pregnant control; NPAzi, non-pregnant rats exposed to azithromycin; PAzi, pregnant rats exposed to azithromycin; NPAmx, non-pregnant rats exposed to amoxicillin; PAmx, pregnant rats exposed to amoxicillin; NPCef, non-pregnant rats exposed to cefaclor; PCef, pregnant rats exposed to cefaclor. * Indicates significant $p < 0.05$.

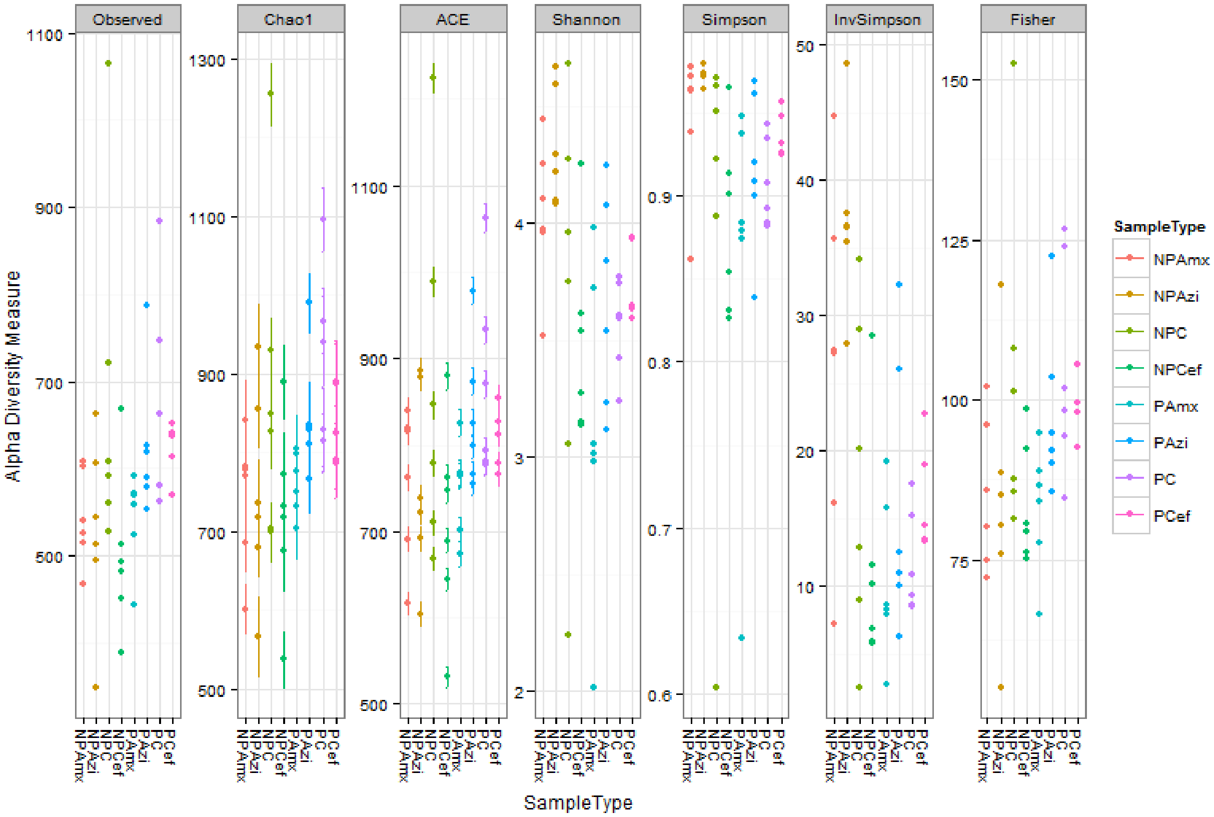

Figure S3: Alpha diversity analysis. NPC, non-pregnant control; PC, pregnant control; NPAzi, non-pregnant rats exposed to azithromycin; PAzi, pregnant rats exposed to azithromycin; NPAmx, non-pregnant rats exposed to amoxicillin; PAmx, pregnant rats exposed to amoxicillin; NPCef, non-pregnant rats exposed to cefaclor; PCef.

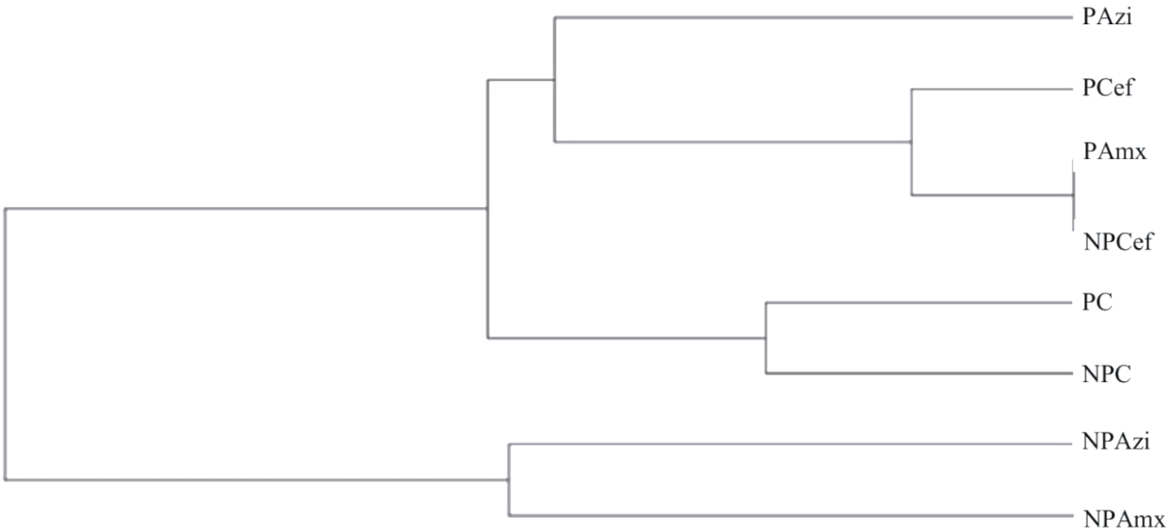

Figure S4: UPGMA distance metric clustering based on taxonomic count. All the groups are cladded in two groups. Comparatively, all the pregnant groups are closely linked except NPCef – which is the most distinct group from the rest non-pregnant groups. NPC, non-pregnant control; PC, pregnant control; NPAzi, non-pregnant rats exposed to azithromycin; PAzi, pregnant rats exposed to azithromycin; NPAmx, non-pregnant rats exposed to amoxicillin; PAmx, pregnant rats exposed to amoxicillin; NPCef, non-pregnant rats exposed to cefaclor; PCef.

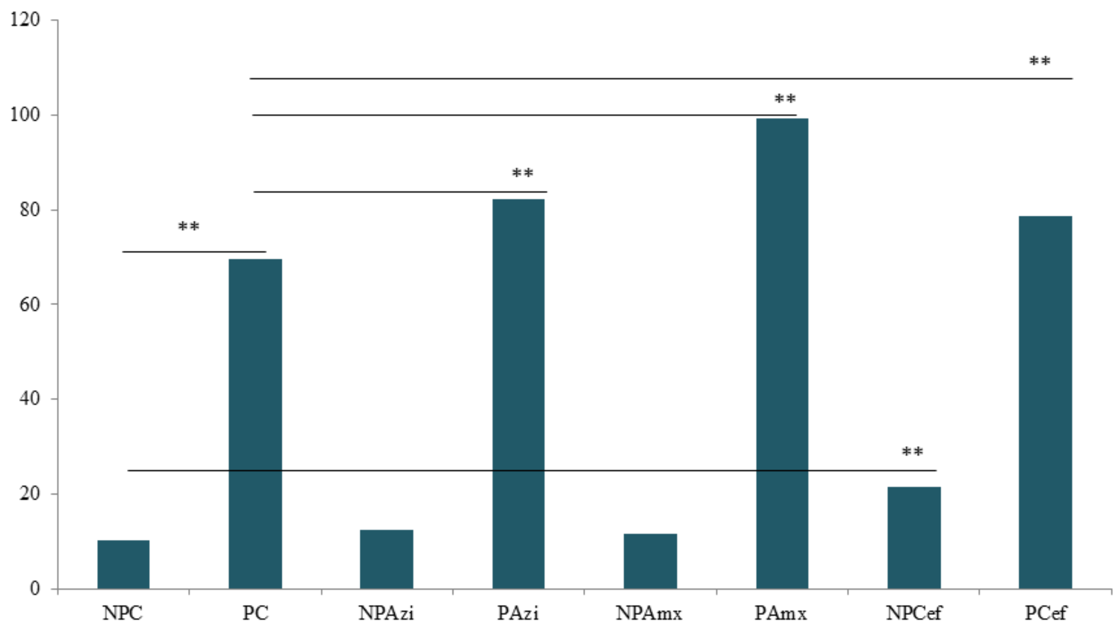

Figure S5: Weight gain in pregnant and non-pregnant rats groups with antibiotic treatments. Statistically, significant weight gain was observed in non-pregnant rats treated with cefaclor, and in pregnant rats treated with azithromycin, amoxicillin and cefaclor compared with their respective controls. * Indicates significant $p < 0.05$. **Highly significant $p \leq 0.01$.
